# Gefitinib loaded nanostructured lipid carriers: characterization, evaluation and anti-human colon cancer activity *in vitro*

**DOI:** 10.1080/10717544.2020.1754526

**Published:** 2020-04-24

**Authors:** Hafiz A. Makeen, Syam Mohan, Mohamed Ahmed Al-Kasim, Ibraheem M. Attafi, Rayan A. Ahmed, Nabeel Kashan Syed, Muhammad Hadi Sultan, Mohammed Al-Bratty, Hassan A. Alhazmi, Mohammed M. Safhi, Raisuddin Ali, M. Intakhab Alam

**Affiliations:** a Department of Clinical Pharmacy, College of Pharmacy, Jazan University, Jazan, Saudi Arabia;; b Substance Abuse and Toxicology Research Centre, Jazan University, Jazan, Saudi Arabia;; c Department of Pharmacology, College of Pharmacy, Jazan University, Jazan, Saudi Arabia;; d Poison Control and Medical Forensic Chemistry Centre, General Directorate of Health Affairs, Jazan, Saudi Arabia;; e Department of Pharmaceutics, College of Pharmacy, Jazan University, Jazan, Saudi Arabia;; f Department of Pharmaceutical Chemistry, College of Pharmacy, Jazan University, Jazan, Saudi Arabia;; g Department of Pharmaceutics, College of Pharmacy, King Saud University, Riyadh, Saudi Arabia

**Keywords:** Gefitinib, Tween 80, nanostructured lipid carriers, sodium lauryl sulfate, MTT assay, colorectal cancer

## Abstract

NLC containing Gefitinib (NANOGEF) was prepared using stearic acid, sesame oil and surfactants (sodium lauryl sulfate and tween 80). NANOGEFs were evaluated for particle size, polydispersity index (PdI), zeta potential, entrapment efficiency (EE), stability, release studies and cytotoxicity studies (MTT assay). The optimized NANOGEF exhibited particle size of 74.06 ± 9.73 d.nm, PdI of 0.339 ± 0.029 and EE of 99.76 ± 0.015%. The TEM study revealed spherical shape of NANOGEF formulations. The slow and sustained release behavior was exhibited by all NANOGEFs. The effects of surfactants were observed not only on particle size but also on zeta potential, entrapment efficiency, stability and release studies. The MTT assay revealed 4.5 times increase in cytotoxicity for optimized NANOGEF (IC_50_ = 4.642 µM) when compared with Gefitinib alone (IC_50_ = 20.88 µM in HCT-116 cells). Thus NANOGEF may be considered as a potential drug delivery system for the cure of colon cancer.

## Introduction

Nano-carriers including lipid nano-carriers exhibited a massive potential for effective delivery of drugs in various diseases, including cancer. The suitability of lipid nano-carriers in the cure of cancer can be explained based on the advantages over conventional chemotherapy (e.g. adverse effect and drug resistance) as well as the advantages associated with specific properties of lipid nano-carriers (improved pharmacokinetics and pharmacodynamics profile of drug, tumor specific drug accumulation, enhance the internalization and intracellular delivery of drugs and reduced biodistribution thereby decreasing the adverse effects of anti-cancer molecules; Rizwanullah et al., 2016; Yingchoncharoen et al., 2016). Nanostructured lipid carrier (NLC) is a novel nano-lipid carrier that has extensively been explored for the delivery of a number of therapeutic agents including duloxetine (Alam et al., 2014), tamoxifen (Shete et al., 2014), paclitaxel (Yang et al., 2013), celecoxib (Patlolla et al., 2010), doxorubicin (Taratula et al., 2013), and curcumin (Madane & Mahajan, 2016). NLCs are considered to be a promising strategy for the delivery of drugs because of their rapid uptake, biodegradability and bioacceptability (Madane & Mahajan, 2016). These are well tolerated lipid carriers in living systems because they are made up of physiological compounds. Moreover, the risk of toxicity is reduced because of the existence of metabolic pathway (Alam et al., 2012). NLC is composed of solid lipid (fat) and liquid lipid (oil). After melting of solid lipid, the liquid lipid is mixed with it. NLCs are considered to be a new generation of solid lipid nanoparticles (SLN) which combine the advantages in addition to overcome the drawbacks associated with SLN. NLC gives better controlled release characteristics, increased drug loading capacity and decreased burst release (Alam et al., 2013). The improved drug delivery characteristics of NLC over SLN can be attributed to the presence of liquid lipid (oil; Saedi et al., 2018).

Colorectal cancer is the third most diagnosed cancer in the western world. In the USA alone, 135000 new cases were diagnosed and nearly 50000 death were reported in the year 2017 (Siegel et al., 2017). Important advancements in the treatment of colorectal cancer have been achieved over the past two decades, increasing our understanding of the disease biology and mechanisms of tumor progression, and advancing early detection and multimodal care (Loree & Kopetz, 2017). Thus, the rate of incidence and mortality of colorectal cancer have been low compared to earlier days (Siegel et al., 2017). Among the cancer pathways, epidermal growth factor receptor (EGFR) plays a crucial role in the carcinogenesis, invasion and metastasis of colorectal cancer (Huang et al., 2017). Gefitinib (GEF) is a selective inhibitor of EGFR tyrosine kinase and it has been widely used in the treatment of lung cancer, either as monotherapy or in combination with other agents (Chang et al., 2018). In colorectal cancer, GEF has been significantly reduced cell proliferation, colony formation and migration in vitro (Huang et al., 2017). Clinical trial conducted with GEF as single or combination therapy showed promising results in colorectal cancers (Gelibter et al., 2007). Like all other cytotoxic drugs, GEF also carries some side effects regardless of its benefits in treating colon cancer (Hartmann et al., 2009). Hence, any attempt in reducing the dose of GEF would be a great achievement in reducing the comorbidities associated with its use. In this study, we hypothesized that the NLC loaded with GEF may exert high sensitivity in cancer cells with low dose. Hence, the objective of this study was to develop and evaluate NLC containing GEF (NANOGEF). The prepared formulations were characterized by the determination of particle size (PS), polydispersity index (PdI), zeta potential (ZP), entrapment efficiency (EE), release behavior and stability studies. In addition, its efficacy in inducing cytotoxicity also has been determined in vitro.

## Materials and methods

Gefitinib was purchased from LC Laboratories (Woburn, MA, USA). Stearic acid (SA) and sodium lauryl sulfate (SLS) were obtained from Himedia (Mumbai, India). Sesame oil (pure) was obtained from the local merchant (Jazan, Saudi Arabia). Tween 80 (T80) was obtained from Loba Chemei (Mumbai, India). RPMI-1640 medium, FBS and penicillin-streptomycin was purchased from Gibco (Invitrogen Corp., USA). Neutral red was purchased from Sigma Chemical Co. (St Louis, MO, USA). All other chemicals and reagents used were of analytical grade.

### Preparation of NLC

Gefitinib loaded NLC (NANOGEF) was prepared by hot homogenization method. Accurately weighed amount of stearic acid (SA; 500 mg) was taken in a beaker and kept on a hot plate for melting. Sesame oil (SO; 250 µl) was measured and transferred to the beaker and mixed (lipid phase). Gefitinib (GEF; 50 mg) was mixed with the lipid phase. Another beaker containing purified water (25 ml) was mixed with tween-80 (T80) and/or sodium lauryl sulfate (SLS; Table 1) and heated up to 70° C (aqueous phase). It was followed by mixing of the aqueous phase with the lipid phase by a homogenizer (HG-15D, WiseTis, Germany) for 20 min at a speed of 5000 rpm.

**Table 1.** Formula for the preparation of different NANOGEF formulations.

Formulation Code	Sodium lauryl sulfate (SLS) (mg)	Tween 80* (T80) (µl)
TNG	–	100
SNG	75	–
STNG	50	25
TSNG	25	75

*Density = 1.07 g/cm^3^.

### Entrapment efficiency (EE)

Amount of GEF that was incorporated into NLC can be differentiated by free GEF by the estimation of EE. The entrapment efficiency in percentage (% EE) is defined as the amount of GEF in percentage incorporated in NLC with respect to the total amount of GEF added in the formulation. The entrapment efficiency of NANOGEF was determined by ultrafiltration centrifugation method (Intakhab Alam et al., 2011). The NANOGEF formulation (2 ml equivalent to 4 mg of GEF) was taken in ultrfiltration tube (Amicon Ultra – 2 ml 3 K, Millipore Ireland) and centrifuged for 15 min at a speed of 4000 *g* (6861 rpm). The filtrate obtained (1 ml) after centrifugation was used for the estimation of free amount of GEF. The filtrate was diluted appropriately and estimated spectrophotometrically at 252 nm using a UV-visible spectrophotometer (UV, Shimadzu, Kyoto, Japan). The following formula was used for EE determination;

$$\mathrm{EE}\;(\%)=\frac{\text{Amount of GEF added }-\text{ Amount of GEF in filtrate}}{\text{Amount of GEF added}}\;\times 100$$

### Particle size, polydispersity index and zeta potential

The mean particle size and polydispersity index (PdI) of NANOGEF was determined by photon correlation spectroscopy with the help of Malvern zetasizer (Nano ZS90, UK). Formulations were appropriately diluted with Millipore water before the analysis of size distribution. Dilution gives appropriate concentration of particles to avoid multiscattering events. The zeta potential was determined to measure the surface charge of NANOGEF with the help of same Malvern zetasizer. All measurements were performed in triplicate.

### Morphology study by transmission electron microscopy (TEM)

The morphology of NANOGEF formulations was studied using the electron microscope (JEOL, JEM 1010, Japan), working in the transmission mode (TEM). The imaging of each sample was done by dispersing a drop of NANOGEF formulations on a copper grid (400 mesh Copper grid with support film carbon type-B; Ted Pella inc., USA).

### Release study

The release study of NANOGEF was performed using dialysis bag (Spectra/Por^(R)^, 12-14 KD MWCO, Spectrum Laboratories, Inc. CA, USA) method. Each formulation (volume equivalent to 4 mg GEF) was transferred to the bag. Both the ends of the bag were tied with thread after filling with formulation. The filled bag was transferred to a beaker containing release media of 100 ml consisting of phosphate buffer solution (pH 7.4) and ethanol (50:50; Emeje et al., 2008). Sampling (2 ml) was done after 0.5, 1, 2, 3, 4, 5, 6, 12, 24 h. Fresh media was replaced in the beaker to maintain the volume of release media and sink condition throughout the experiment. The aliquot of sample was filtered through a 0.45 µm filter using a syringe. The collected samples were measured by UV-spectrophotometer (Schimadzu, Japan) at a λ_max_ of 252 nm.

### Stability studies

The stability studies of NANOGEF formulations were performed to assess the effect of temperature. The NANOGEF formulations were stored and analyzed after three months of storage at room temperature (25 °C). The effect of temperature was assessed in terms of particle size, polydispersity index (PdI) and entrapment efficiency (% EE).

### Cell line and cell cultures

The human colon cancer cells HCT 116 were obtained from ATCC. The cell line was grown and maintained in RPMI-1640 medium, pH 7.4. The media were supplemented with FBS (10%), penicillin (100 U/ml), streptomycin (100 g/ml), and cells were grown in CO_2_ incubator (New Brunswick Scientific) at 37 °C with 90% humidity and 5% CO_2_. Cells were treated with drugs which is dissolved in DMSO (DMSO <0.05% in media), while the untreated control cultures received only the vehicle (DMSO < 0.05% in media).

### Cell viability assay

The cytotoxicity profiles of the formulations were assessed using the 3-(4, 5-dimethylthiazol-2-yl)-2, 5-diphenyltetrazolium bromide (MTT) microculture tetrazolium viability assay (Syam et al., 2012). Briefly, the various concentrations of samples (highest was 200 µg/ml) were plated out in triplicates and incubated for 48 h. Each plate included untreated cell controls and a blank cell-free control. After incubation, MTT (5 mg/ml) was added to each well and the plates were incubated for further 4 h after which the media was removed. DMSO (100 µl) was added into each well to solubilize the formazan crystals. The absorbance was read at wavelength of 490 nm using a microtiter plate reader (BioTek Instruments, Winooski, VT, USA).The percentage cellular viability was calculated with the appropriate controls taken into account. The experiment was done in triplicate. The inhibitory rate of cell proliferation was calculated by the following formula:

$$\text{Growth inhibition}=\;\frac{\text{OD control}-\text{OD treated}}{\text{OD control}}\;\times 100$$

The cytotoxicity of sample on cancer cells was expressed as IC_50_ values (the sample concentration reducing the cell count of treated cells by 50% with respect to untreated cells).

### Morphological changes in treated cells

HCT-116 cells were grown on 24 well culture plates and incubated overnight. The cells were then treated in duplicates (2 batches) with formulations at IC_50_ and kept for 48 h. After the treatment, medium was removed and cells were washed with cold sterile PBS. Neutral red (0.5%) was then added at a volume of 100 µl to each well and stained for 2 minutes. The stains were washed out with PBS and immediately observed under an inverted microscope (Latorre et al., 2019).

### Statistical analysis

All data presented as means ± SD. One way ANOVA was used for statistical analysis and p value less than 0.05 (*p* < .05) was considered as statistically significant difference.

## Results and discussions

NANOGEF was successfully prepared by hot homogenization method (Intakhab Alam et al., 2011) using the two lipids (fat and oil) and two surfactants (SLS and T80). The formula for the preparation of NANOGEF was optimized based on particle size, zeta potential, uniformity of dispersion and stability studies. The process parameters including speed and duration of homogenization were optimized to 5000 rpm and 20 min respectively.

### Particle size, polydispersity index and zeta potential

**Figure 1.**
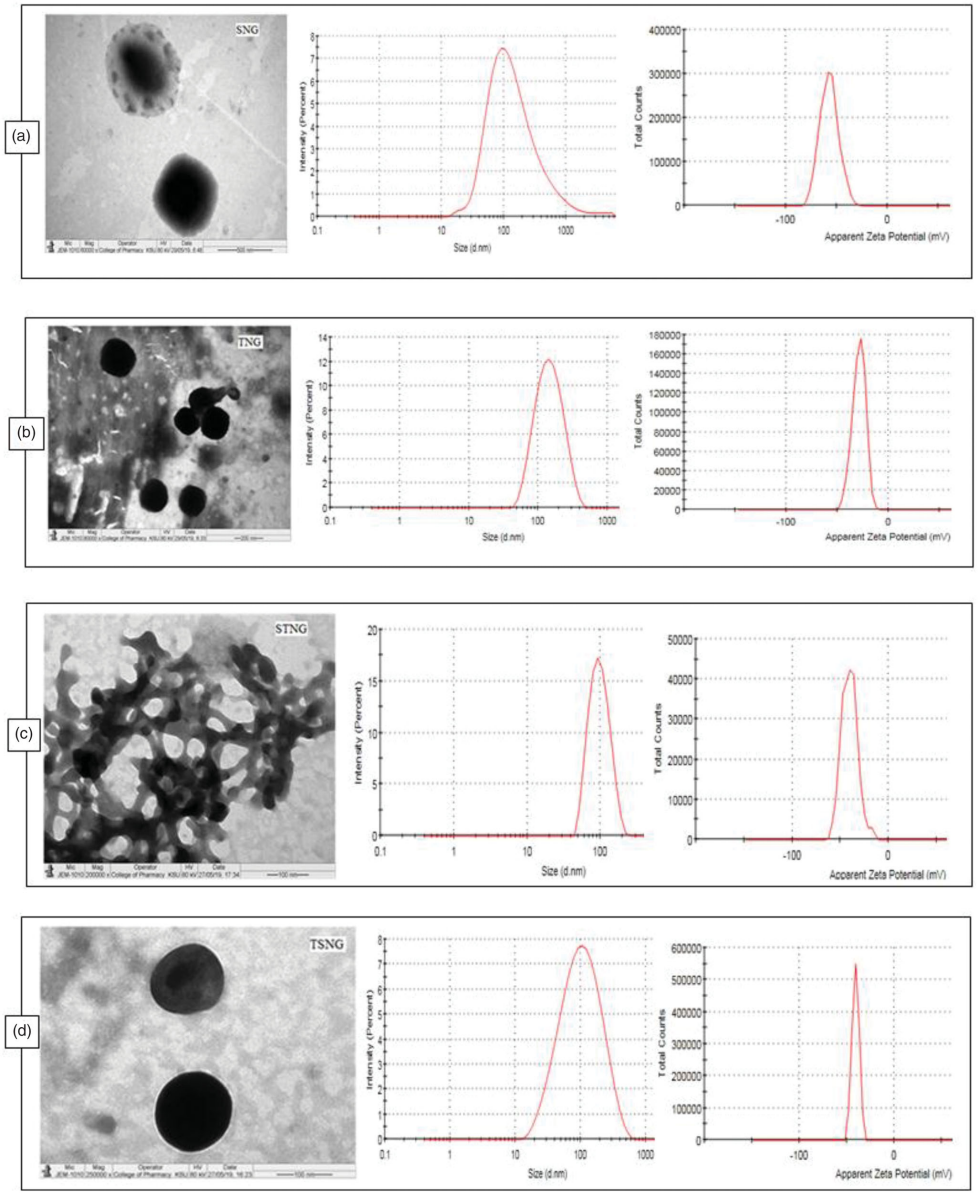
TEM, size and zeta potential of SNG (a) (size = 102.6 d.nm, ZP = −57.3 mV); TNG (b) (size = 127 d.nm, ZP = −28.9 mV); STNG (c) (size = 92.72 d.nm, ZP = −40.2 mV); TSNG (d) (size = 82.52 d.nm, ZP = −40.6 mV). (*n* = 1).

NANOGEF was prepared and analyzed in order to determine their particle size distribution, zeta potential and PdI values. The mean size was found to be 129.7, 108.9, 96.7 and 74.06 nm for SNG, TNG, STNG and TSNG respectively (Table 2; Figure 1). The effect of surfactants on particle size of NLC containing GEF can be witnessed. The size of NLC prepared using SLS was found to be larger than the NLC prepared using T80. The effect of T80 on size reduction of NANOGEF was observed to be more than SLS (i.e. SNG > TNG). The size of NANOGEF was further reduced when both the surfactants (T80 and SLS) were used together. Moreover, the combined effect of surfactants on size reduction was observed to be more in TSNG than STNG. Thus, the combined effect of two surfactants was found to be more noticeable than the use of single surfactant. The literature review shows that type of surfactants and their concentrations greatly affect the size of NLC. Kaur et al. (2016) obtained NLC loaded with paclitaxel of different size under the influence of different concentrations of tween 20 (T20), tween 60 (T60) and T80 and investigated that surfactant concentration is considered to be one of the most effective factor affecting the size and PdI. Particle size greatly affects the cellular uptake by tumor cells. It is considered one of important parameters for passive targeting of anticancer agents to tumors (Mozafari et al., 2009). Thus the particle size ranging between 50 and 200 nm of nanocarriers is considered to be effectively suitable for delivery of chemotherapeutic agents. The size of nanocarriers between 50 and 100 nm is investigated to be suitable for avoidance of monocellular phagocytic system (MPS) uptake and prolonged blood circulation time. Moreover, the nanocarriers of size smaller than 150 nm (or 200 nm) are found to be effective for anticancer treatment by passive targeting to tumor tissues via enhanced permeability and retention (EPR; Danaei et al., 2018). Moreover, the type of cell lines significantly affecting the recruitment of nanocarriers of different sizes. For example, internalization of nanocarriers of size 93–220 nm is investigated to be preferred by hepatic cell lines of Hep G2 and Hepa 1-6 type; however, the nanocarriers dimension up to one micron is preferred by HUVEC and ECV 30 cell lines (Şenel, 2019).

**Table 2.** Results of characterization (size, polydispersity index – PdI, zeta potential, entrapment efficiency – EE) of NANOGEFs.

Formulation Code	Size (d.nm) (n = 3) (±SD)	PdI (n = 3) (±SD)	Zeta Potential (mV) (n = 3) (±SD)	EE (%) (n = 3) (±SD)
SNG	129.70 ± 25.69	0.386 ± 0.043	−57.3 ± 3.9	99.85 ± 0.115
TNG	108.33 ± 22.13	0.193 ± 0.093	−27.23 ± 2.39	99.96 ± 0.033
STNG	96.70 ± 6.76	0.168 ± 0.034	−66.7 ± 5.0	99.66 ± 0.184
TSNG	74.06 ± 9.73	0.339 ± 0.029	−42.7 ± 2.44	99.76 ± 0.015

The polydispersity index (PdI) was perceived to be 0.386, 0.193, 0.168 and 0.339 for SNG, TNG, STNG and TSNG respectively (Table 2). PdI is an indicator of aggregation/agglomeration of particles. When the PdI value is closer to zero, it indicates monodispersity conduct of the system. Higher value of PdI (> 0.5) denotes the polydispersity of the system. The polydispersed systems have greater tendency to aggregation than the monodispersed systems (Jirgensons & Straumanis, 2013).

Zeta potential (ZP) is considered to be a key indicator of physical stability of colloidal dispersions including NANOGEF preparations. Moreover, ZP enhances the capability of nanocarriers to bind with cell membrane. A specific value of zeta potential is therefore necessary to create an electrostatic interaction with cell membranes for the transport of therapeutic substances (Şenel, 2019). Cell membranes do possess positive sites besides negatively charged domains. The negatively charged nanocarriers bind to the positive sites in the form of clusters to counter against the repulsive interaction of large negatively charged domains. This binding leads to localized neutralization and gelation of membranes that consequence in favoring endocytosis for cellular uptake (Patil et al., 2007; Foroozandeh & Aziz 2018).

The higher values of ZP, either positive or negative, the higher will be the tendency to stabilize the colloidal preparations. Typically, the aggregation of particles is prevented for the formulations containing charged particles with ZP value of (>‖20‖; Gonzalez-Mira et al., 2011). This much of ZP is sufficient to maintain the electrostatic repulsion among the similarly charged particles which prevent the aggregation. ZP of NANOGEF is the potential difference between NLC surface and its liquid medium (i.e. particle–liquid interface). All NANOGEF formulations were determined to have negative charge (negative ZP). The slightly ionized fatty acids may likely contribute to negative charges of NANOGEF formulations (Sanad et al., 2010). The ZP of NANOGEF formulations was largely affected by the type of surfactants used. SLS containing formulations was found to exhibit more value of ZP than T80 containing formulations (i.e. SNG > TNG; Table 2). Likewise, the ZP was also affected by the quantity of surfactants used for the preparation of NANOGEF formulations (i.e. STNG > TSNG). The combined effect of both the surfactants (SLS and T80) was found to be more on ZP than these were used alone. Moreover, SLS gives more stable preparations of NANOGEF when used in higher quantity with T80. Thus, STNG is more stable electrostatically than TSNG based on the value of ZP. The lowering value of ZP of the formulations (TSNG and TNG) containing nonionic surfactants including T80 may be explained on the basis of its nonspecific adsorption which shields the expression of electrostatic charges on NANOGEF formulations (How et al., 2013). Moreover, the higher ZP value of TSNG than TNG may be explained on the basis of availability of space on the surface of NANOGEF particles for adsorption. Thus, the spaces available for adsorption on NANOGEF in case of TSNG for T80 were less due to the presence of SLS which resulted in higher ZP value. Similarly, more spaces were available in TNG for T80 adsorption which resulted in lower ZP value than TSNG. Consequently, the order of stability can be arranged as STNG > SNG > TSNG > TNG.

### Entrapment efficiency (EE)

The entrapment efficiency (EE) of all NANOGEF preparations was found to be between 99.66 ± 0.184% and 99.96 ± 0.033 (Table 2). The high EE (%) of NANOGEF preparations may be attributed to the solubility of GEF in solid lipid (stearic acid) and liquid lipid (sesame oil). Furthermore the partition of GEF between oil phase and aqueous phase may contribute to EE value.

The incorporation of liquid lipid into solid lipid increases the imperfection in the crystal lattice and reduction of crystallinity with high drug compatibility, helping to accommodate more amount of GEF and resulting in high value of EE. Thus, GEF molecules can be accommodated in between fatty acid chains and/or lipid layers. Similar findings were observed earlier (Intakhab Alam et al., 2011; Zhang et al., 2013; Jain et al., 2015; Aslam et al., 2016; Thang et al., 2017).

The concentration of surfactants may also contribute to high value of EE. The addition of surfactants increases the viscosity of aqueous phase thereby decreasing the diffusion speed of GEF which results in high value of EE. The increased surface area of NANOGEF was obtained because of smaller size of particles where GEF molecules were incorporated. It may happen due to the obtainability of enough surfactant which enables GEF to remain incorporated within the particle and results in high value of EE. The impact of surfactant concentrations on the EE values were found to be in accordance to the findings of others (Jain et al., 2015; Aslam et al., 2016); Zhang et al. (2013).

### Morphology study

The morphology of all NANOGEFs was studied by TEM images as shown in Figure 1. All NANOGEFs were found to have almost spherical and uniform shape with nanometer range size. As per images, no visible aggregation of particles was observed except STNG. The aggregation in STNG may be due to insufficient drying time given to samples before TEM analysis.

### Release study

**Figure 2.**
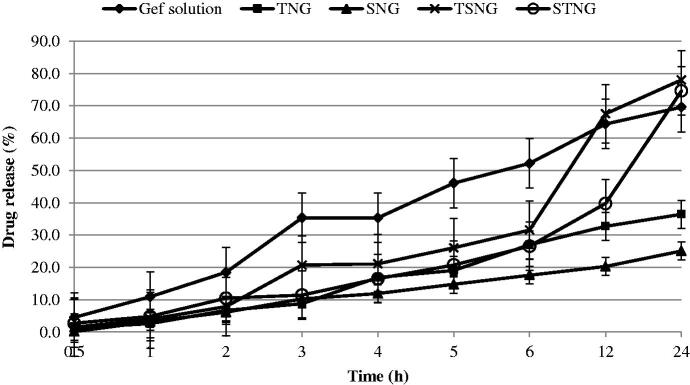
Release pattern of NANOGEF formulations. TNG and SNG exhibited lesser amount of GEF release than TSNG and STNG (*p* < .05).

The in vitro release studies of NANOGEF formulations were performed by the dialysis bag diffusion technique. The study was performed under sink conditions to avoid interference from GEF solubility in the release medium. The release pattern of NANOGEF formulations has been shown in Figure 2. All NANOGEF formulations exhibited slow and sustained release of GEF, as expected for NLC formulations. It may be attributed to the solid matrix of NLC and the subsequent immobilization (Gonzalez-Mira et al., 2011). In addition, the sustained release behavior of lipid nanocarriers including NLC may be explained on the basis of release of active ingredients from within the NLC core, partitioning between the water and the lipid matrix, as well as barrier function of the interfacial membrane (Roohinejad et al., 2018). Moreover, the homogeneity of entrapment of drug throughought the system may contribute to the slow release of drug from lipid nanocarriers (Sanad et al., 2010). The high value of EE (>99%) supports the release characteristics of NANOGEF formulations. Thus, NLC may be considered to be suitable vehicle for the delivery of GEF where a single dose would be sufficient for prolonged action on cancer cells.

The burst effect was not observed in the release pattern. The EE value of NANOGEF formulations was found to be almost hundred percent (>99%) and unentrapped amount of GEF was found to be almost zero percent which is evidenced by the release studies too. Generally, the burst effect in release studies is obtained due to the presence of unentrapped drug on the surface of NLC (Intakhab Alam et al., 2011; Kaur et al., 2016). Our release studies exhibited absence of burst effect which is only possible in case when there is unentrapped GEF in negligible amount.

The GEF solution was prepared by dissolving in water containing dimethylsulfoxide (0.2% v/v). The GEF solution exhibited faster release than NANOGEF preparations. The release of GEF from GEF solution was sustained by dialysis membrane only.

However, the sustained release behavior of NANOGEF formulations may be attributed to both dialysis membrane as well as NLC. TNG and SNG formulations exhibited lesser amount of GEF released after 24 h of study than the amount released from TSNG and STNG (*p* < .05). This is explained on the basis of presence of single surfactant in both TNG and SNG formulations. Moreover T80 seems to be more effective than SLS for the release of GEF from NANOGEF formulations (TNG > SNG). Correspondingly the combined effect of T80 and SLS on the release behavior of GEF was more noticeable in TSNG than STNG (TSNG > STNG). Surfactant action for longer duration of time (sampling after 24 h; a gap of 12 h) in NANOGEF formulations and accumulation of released GEF may be considered a responsible factor for more amount of released GEF than in case of GEF solution.

Furthermore, the mechanism of release of GEF from NANOGEFs was assessed. The release data of NANOGEFs were fitted into different kinetic models (Table 3). Based on the higher value of correlation coefficients (R^2^), the preferred model that fits best to the release data was zero order (concentration independent release rate) for SNG (R^2^ = 0.976) and TNG (R^2^ = 0.994) and Korsmeyer–Peppas model (anomalous diffusion) for STNG (R^2^ = 0.993) and TSNG (R^2^ = 0.959). Thus the combined effect of two surfactants (T80 and SLS) was observed in the mechanism of release of GEF from NANOGEFs.

**Table 3.** Correlation coefficients (R^2^) value for GEF release from different NANOGEFs.

Order of reaction	SNG	TNG	STNG	TSNG
Zero	0.976	0.994	0.777	0.858
First	0.920	0.683	0.967	0.924
Higuchi	0.890	0.910	0.971	0.954
Korsmeyer–Peppas model	0.923	0.759	0.993	0.959

### Stability studies

**Figure 3.**
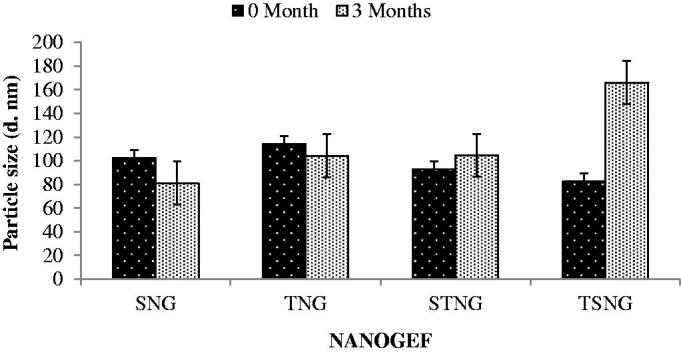
Effect of storage temperature on particle size of NANOGEFs. Storage temperature decreased particle size (*p* > .05) in case of SNG and TNG (may be because of complete covering of surface by surfactant leads to decrease in size during storage period). STNG (*p* > .05) and TSNG (*p* < .05) exhibited increase in particle size.

Temperature is envisaged to be a key factor affecting the stability of nano-formulations including NLCs. Stability at room temperature (25 °C) is imagined to be worthy as it does not require any special condition for the storage of formulations. Thus, the NANOGEF formulations were stored at room temperature for three months and evaluated for particle size, polydispersity index (PdI) and entrapment efficiency (% EE). The effect of storage conditions was observed not only on particle size but also on PdI and EE of all NANOGEF formulations. The effect on particle size was perceived as shown in Figure 3. The storage condition favors the particle growth in case of TSNG and STNG. However, TSNG exhibited larger particle size than STNG (*p* < .05). Nevertheless, the particle size of TSNG remained in colloidal nanometer range (<550 nm; Elmowafy et al., 2017). The increase in particle size of STNG was found to be lower than two folds suggesting the absence of aggregation. Such type of changes may be attributed to the swelling or adsorption of additional surfactants on the surface of NLC (Saedi et al., 2018). Furthermore, the storage period affected the size of SNG and TNG formulations. Their particle size after storage was observed to be less than before storage (*p* > .05). The decrease in size of NLCs (SNG and TNG) during storage period of three months may be explained on the basis of covering of surface completely by the molecules of surfactants. The larger particle size of NANOGEF before the storage period may be because of incomplete covering of surface by surfactants. The storage period provides sufficient interval for surfactants to enter the surfaces to cover it completely.

**Figure 4.**
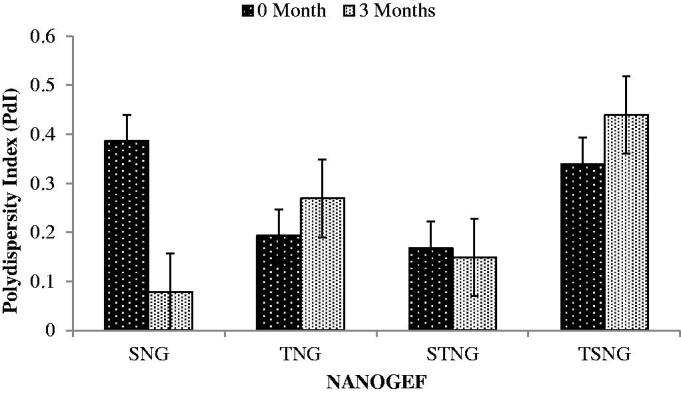
Effect of storage temperature on polydispersity index (PdI). SNG (*p* < .05) and STNG (*p* > .05) exhibited lowering in PdI. TNG and TSNG exhibited increase in PdI (*p* > .05).

PdI is considered to be an important parameter for physical stability of nanoformulations. Smaller value of PdI is desirable for long term stability of nanosuspensions (Shimojo et al., 2019). Effect of storage period on PdI has been shown in Figure 4. NANOGEF formulations including SNG (*p* < .05) and STNG (*p* > .05) exhibited decrease in PdI value; however TNG and TSNG formulations revealed increase in the value (*p* > .05). Based on this concept SNG is considered to be most stable on long term stability study followed by STNG, TNG and TSNG (i.e. SNG > STNG > TNG > TSNG).

**Figure 5.**
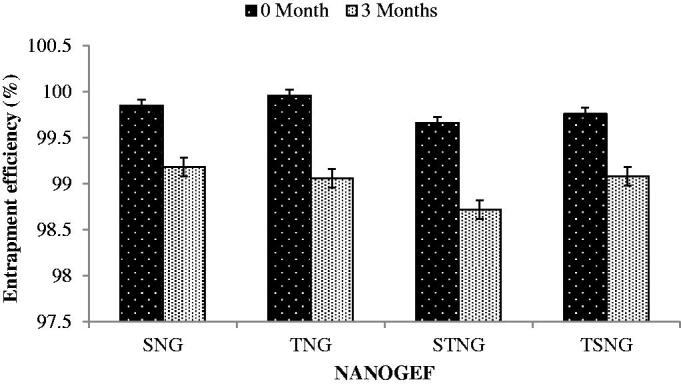
Effect of storage temperature on entrapment efficiency (% EE). All NANOGEFs were estimated to show decrease in EE (*p* > .05).

The NANOGEF formulations exhibited decrease in EE during storage for three months (Figure 5). The decrease in EE was estimated to be less than 1 percent (<1%) in all formulations (*p* > .05). Thus the NANOGEF formulations exhibited admirable ability to reduce the expulsion of GEF during the storage of three months at room temperature (25 °C).

**Figure 6.**
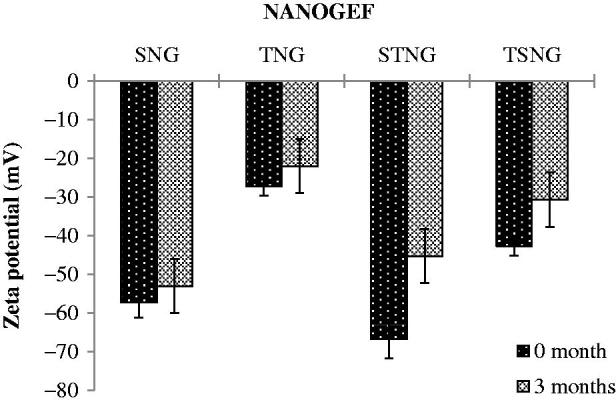
Effect of storage temperature on zeta potential. All NANOGEFs were found to exhibit decrease in ZP (*p* > .05).

The ZP value of NANOGEF formulations decreased when determined after the completion of storage period (Figure 6). The decrease in ZP value was non-significant (*p* > .05). The electrostatic layer was maintained around the nanoparticles throughout the system. Thus, the NANOGEFs exhibited a good physical stability even after three months of storage. The freshly prepared nano-dispersions exhibit maximum value of zeta potential followed by a decline in value with the increase of lapsed time. The decrease in ZP value of NLC dispersions may be explained on the basis of property of particles to agglomerate over storage for a period of time. The agglomeration tendency of particles decreases the dispersion force between particles and charges over the particles (Nakatuka et al., 2015).

### Cytotoxicity study

**Figure 7.**
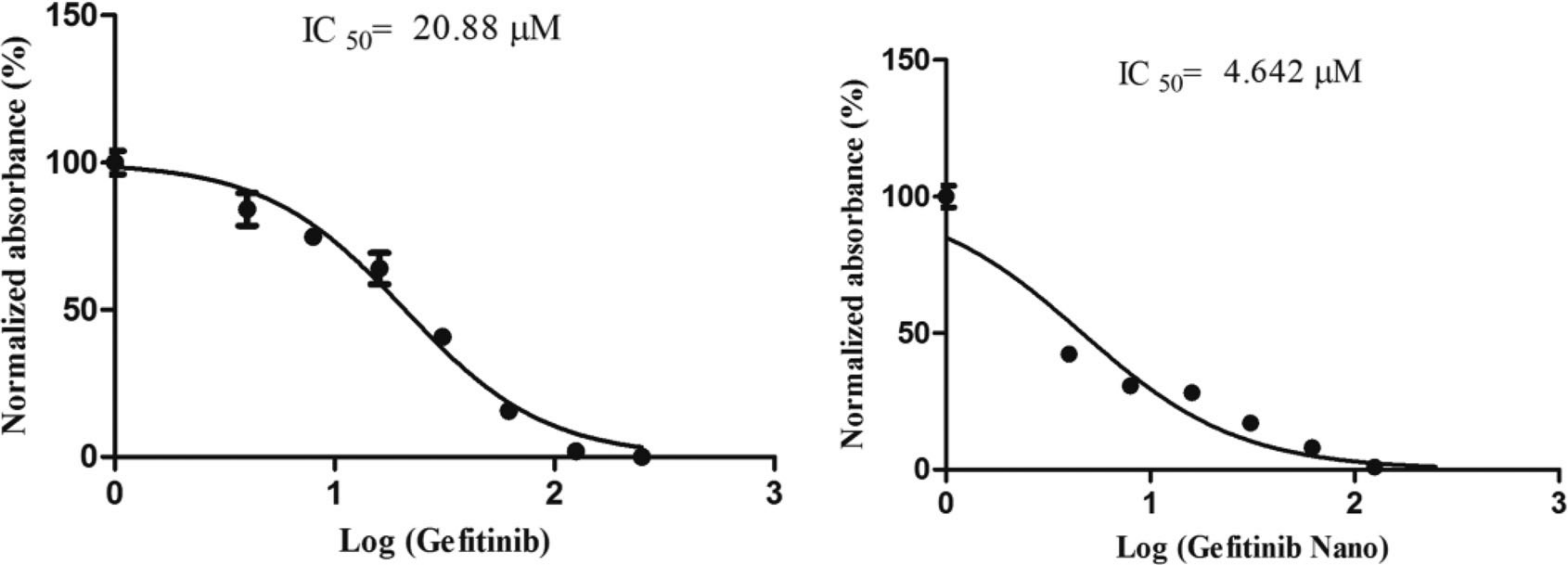
Inhibition effect of GEF alone and NANOGEF (TSNG) (*p* < .05) on HCT 116 cells. Results are presented with means ± SD for *n* = 3.

The cytotoxicity assay (MTT) was performed to reveal the anticancer potential of NANOGEF. The cytotoxicity study of optimized NANOGEF (i.e. TSNG – Gefitinib Nano) was performed and compared with GEF alone. GEF alone exhibited significant cytotoxicity with an IC_50_ of 20.88 µM; however TSNG (Gefitinib Nano) revealed 4.5 times increase in the cytotoxicity with IC_50_ 4.642 µM (Figure 7). Moreover, the NLC blank exhibited no cytotoxicity within the range used in this study.

**Figure 8.**
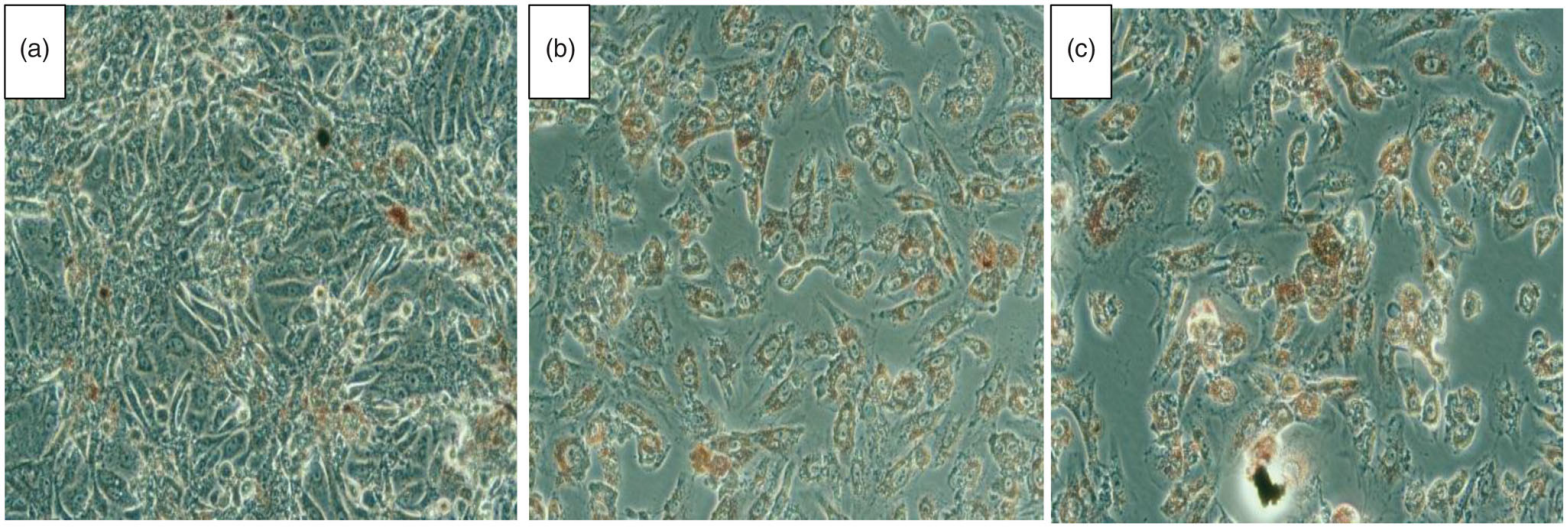
HCT 116 cells treated with IC50 concentration of GEF (B), TSNG (C) and Control (A). The red color inside the cells indicates the infiltrated neutral red in dead cells.

Furthermore, the morphological study confirms the cytotoxicity observed in the MTT assay. Neutral red is a dye which is readily taken only by dead cells. Hence as shown in Figure 8, a significant uptake of the dye indicates that NANOGEF (TSNG) exhibited similar cytotoxicity in the cancer cells at very low dose.

In the clinical practice, cytotoxic drugs exhibit various practical issues such as high toxicity, poor specificity in cancer cells and resistance to pharmaceutical formulations. Additionally, the high affinity of conventional chemotherapeutic drugs with blood serum also affect their bioavailability in greater extend (Mayur et al., 2009). All these factors made the rise in concern over the use of cytotoxic drug which tend to induce high toxicity and reduced efficacy. Additionally it is to be noted that cellular pharmaceutical resistance is an important event in reducing the efficacy of cytotoxic agents (Roberti et al., 2006). Multidrug resistance phenotypes always had showed an efflux of cytotoxic agents out of the cytoplasmic membrane through transporters. Moreover, the special characteristics of highly cancerous tumor cells are inclined toward resistance of cytotoxic drugs via decreasing high intra-tumoral pharmaceutical concentration in solid tumors. It has become a tough challenge and dilemma with practitioners to select among the high dose with better efficacy and low dose with lesser side-effects. Nanoparticle drug delivery system has gained fabulous consideration in cancer research which offers a great leap in managing such obstacles of cancer treatment. These carriers offers improved drug absorption, higher uptake of drug by cancerous cells, enhanced intracellular penetration and prevention of interaction with biological environments such as plasma binding. In our study, the NANOGEF have shown significant difference in the dose of cytotoxicity. This phenomenon may be due to the increased uptake of NANOGEF, cellular concentrations and the significant presence of EGFR in colorectal cancers. Meanwhile, GEF alone correlated with its low level of cytotoxicity to HCT-116 cells.

## Conclusion

Gefitinib loaded NLC (NANOGEF) was successfully prepared by hot homogenization method using fat (stearic acid), oil (sesame oil) and surfactants (SLS and T80). NANOGEF formulations exhibited admirable characterization parameters including particle size, polydispersity index, zeta potential, entrapment efficiency, TEM and release studies. Based on the parameters evaluated for NANOGEF formulations TSNG may be considered to be the best formulation among all. The lipid nature and the smaller size (<100 nm) ensure sufficient permeation of GEF through biological barriers which may result in satisfactory therapeutic consequences. The release behavior endorses prolonged action on the site of target. Together, with high EE value (>95%) of TSNG may be considered to be positive factors for enhanced therapeutic efficacy.

In this study we also revealed its in vitro potential for the treatment of colorectal cancer too (It was approved for lung cancer treatment by FDA). Furthermore, the anticancer effect of GEF was investigated to be 4.5 times better when loaded in NLC. In future GEF loaded NLC may be used for the treatment of colorectal cancer with reduced dose thereby reduced exposure of body and hence less adverse effects. Thus, the NANOGEF formulation of TSNG composition may be expected to be used as a drug delivery system for effective therapy of cancer after performing the in vivo studies.

## References

[CIT0001] Alam MI, Baboota S, Ahuja A, et al. (2012). Intranasal administration of nanostructured lipid carriers containing CNS acting drug: pharmacodynamic studies and estimation in blood and brain. J Psychiatr Res 46:1133–8. 22749490 10.1016/j.jpsychires.2012.05.014

[CIT0002] Alam MI, Baboota S, Ahuja A, et al. (2013). Intranasal infusion of nanostructured lipid carriers (NLC) containing CNS acting drug and estimation in brain and blood. Drug Deliv 20:247–51. 23869788 10.3109/10717544.2013.822945

[CIT0003] Alam MI, Baboota S, Ahuja A, et al. (2014). Pharmacoscintigraphic evaluation of potential of lipid nanocarriers for nose-to-brain delivery of antidepressant drug. Int J Pharm 470:99–106. 24810241 10.1016/j.ijpharm.2014.05.004

[CIT0004] Aslam M, Aqil M, Ahad A, et al. (2016). Application of Box–Behnken design for preparation of glibenclamide loaded lipid based nanoparticles: optimization, in vitro skin permeation, drug release and in vivo pharmacokinetic study. J Mol Liq 219:897–908.

[CIT0005] Chang TC, Chin YT, Nana AW, et al. (2018). Enhancement by nano-diamino-tetrac of antiproliferative action of gefitinib on colorectal cancer cells: mediation by EGFR sialylation and PI3K activation. Horm Canc 9:420–32. 10.1007/s12672-018-0341-x PMC6223990 30187356

[CIT0006] Danaei M, Dehghankhold M, Ataei S, et al. (2018). Impact of particle size and polydispersity index on the clinical applications of lipidic nanocarrier systems. Pharmaceutics 10:57. 10.3390/pharmaceutics10020057 PMC6027495 29783687

[CIT0007] Elmowafy M, Ibrahim HM, Ahmed MA, et al. (2017). Atorvastatin-loaded nanostructured lipid carriers (NLCs): strategy to overcome oral delivery drawbacks. Drug Deliv 24:932–41. 28617150 10.1080/10717544.2017.1337823 PMC8241136

[CIT0008] Emeje M, Nwabunike P, Isimi C, et al. (2008). Hydro-alcoholic media: an emerging in vitro tool for predicting dose dumping from controlled release matrices. J of Pharmacology and Toxicology 3:84–92.

[CIT0009] Foroozandeh P, Aziz AA. (2018). Insight into cellular uptake and intracellular trafficking of nanoparticles. Nanoscale Res Lett 13:339. 30361809 10.1186/s11671-018-2728-6 PMC6202307

[CIT0010] Gelibter AJ, Gamucci T, Pollera CF, et al. (2007). A phase II trial of gefitinib in combination with capecitabine and oxaliplatin as first-line chemotherapy in patients with advanced colorectal cancer. Curr Med Res Opin 23:2117–23. 17651538 10.1185/030079907X226113

[CIT0011] Gonzalez-Mira E, Egea M, Souto E, et al. (2011). Optimizing flurbiprofen-loaded NLC by central composite factorial design for ocular delivery. Nanotechnology 22:045101. 21169662 10.1088/0957-4484/22/4/045101

[CIT0012] Hartmann JT, Haap M, Kopp HG, Lipp HP. (2009). Tyrosine kinase inhibitors-a review on pharmacology, metabolism and side effects. CDM 10:470–81. 10.2174/138920009788897975 19689244

[CIT0013] How CW, Rasedee A, Abbasalipourkabir R. (2013). Characterization and cytotoxicity of nanostructured lipid carriers formulated with olive oil, hydrogenated palm oil, and polysorbate 80. IEEE Transon Nanobioscience 12:72–8. 10.1109/TNB.2012.2232937 23268387

[CIT0014] Huang CW, Chen YT, Tsai HL, et al. (2017). EGFR expression in patients with stage III colorectal cancer after adjuvant chemotherapy and on cancer cell function. Oncotarget 8:114663. 29383110 10.18632/oncotarget.23072 PMC5777722

[CIT0015] Intakhab Alam M, Baboota S, Ahuja A, et al. (2011). Nanostructured lipid carrier containing CNS acting drug: formulation, optimization and evaluation. CNANO 7:1014–27.

[CIT0016] Jain K, Sood S, Gowthamarajan K. (2015). Optimization of artemether-loaded NLC for intranasal delivery using central composite design. Drug Delivery 22:940–54. 24512368 10.3109/10717544.2014.885999

[CIT0017] Jirgensons B, Straumanis ME. 2013. A short textbook of colloid chemistry. Burlington (MA): Elsevier.

[CIT0018] Kaur P, Garg T, Rath G, et al. (2016). Development, optimization and evaluation of surfactant-based pulmonary nanolipid carrier system of paclitaxel for the management of drug resistance lung cancer using Box-Behnken design. Drug Deliv 23:1912–25. 25544602 10.3109/10717544.2014.993486

[CIT0019] Latorre A, Latorre A, Castellanos M, et al. (2019). Multifunctional albumin-stabilized gold nanoclusters for the reduction of cancer stem cells. Cancers 11:969. 10.3390/cancers11070969 PMC6678462 31295963

[CIT0020] Loree JM, Kopetz S. (2017). Recent developments in the treatment of metastatic colorectal cancer. Ther Adv Med Oncol 9:551–64. 28794806 10.1177/1758834017714997 PMC5524248

[CIT0021] Madane RG, Mahajan HS. (2016). Curcumin-loaded nanostructured lipid carriers (NLCs) for nasal administration: design, characterization, and in vivo study. Drug Deliv 23:1326–34. 25367836 10.3109/10717544.2014.975382

[CIT0022] Mayur Y, Peters G, Rajendra Prasad V, et al. (2009). Design of new drug molecules to be used in reversing multidrug resistance in cancer cells. Curr Cancer Drug Targets 9:298–306. 19442050 10.2174/156800909788166619

[CIT0023] Mozafari M, Pardakhty A, Azarmi S, et al. (2009). Role of nanocarrier systems in cancer nanotherapy. J Liposome Res 19:310–21. 19863166 10.3109/08982100902913204

[CIT0024] Nakatuka Y, Yoshida H, Fukui K, Matuzawa M. (2015). The effect of particle size distribution on effective zeta-potential by use of the sedimentation method. Adv Powder Technol 26:650–6.

[CIT0025] Patil S, Sandberg A, Heckert E, et al. (2007). Protein adsorption and cellular uptake of cerium oxide nanoparticles as a function of zeta potential. Biomaterials 28:4600–7. 17675227 10.1016/j.biomaterials.2007.07.029 PMC2259388

[CIT0026] Patlolla RR, Chougule M, Patel AR, et al. (2010). Formulation, characterization and pulmonary deposition of nebulized celecoxib encapsulated nanostructured lipid carriers. J Controlled Release 144:233–41. 10.1016/j.jconrel.2010.02.006 PMC2868936 20153385

[CIT0027] Rizwanullah M, Ahmad J, Amin S. (2016). Nanostructured lipid carriers: a novel platform for chemotherapeutics. Curr Drug Deliv 13:4–26. 26279117 10.2174/1567201812666150817124133

[CIT0028] Roberti A, Sala DL, Cinti C. (2006). Multiple genetic and epigenetic interacting mechanisms contribute to clonally selection of drug‐resistant tumors: current views and new therapeutic prospective. J Cell Physiol 207:571–81. 16250021 10.1002/jcp.20515

[CIT0029] Roohinejad S, Greiner R, Oey I, Wen J. 2018. Emulsion-based systems for delivery of food active compounds: formation, application, health and safety. Hoboken (NJ): Wiley Online Library.

[CIT0030] Saedi A, Rostamizadeh K, Parsa M, et al. (2018). Preparation and characterization of nanostructured lipid carriers as drug delivery system: influence of liquid lipid types on loading and cytotoxicity. Chem Phys Lipids 216:65–72. 30219661 10.1016/j.chemphyslip.2018.09.007

[CIT0031] Sanad RA, AbdelMalak NS, elBayoomy TS, Badawi AA. (2010). Formulation of a novel oxybenzone-loaded nanostructured lipid carriers (NLCs). Aaps Pharmscitech 11:1684–94. 21107771 10.1208/s12249-010-9553-2 PMC3011069

[CIT0032] Şenel B. 2019. In vitro preliminary studies of chitooligosaccharide coated nanostructured lipidic nanoparticles for efficient gene delivery. J Res Pharm 23: 671–81.

[CIT0033] Shete HK, Selkar N, Vanage GR, Patravale VB. (2014). Tamoxifen nanostructured lipid carriers: enhanced in vivo antitumor efficacy with reduced adverse drug effects. Int J Pharm 468:1–14. 24704438 10.1016/j.ijpharm.2014.03.056

[CIT0041] Shimojo AAM, Fernandes ARV, Ferreira NRE, Sanchez-Lopez E, Santana MHA, Souto EB. (2019). Evaluation of the Influence of process parameters on the properties of resveratrol-loaded NLC using 22 full factorial design. Antioxidants 8:272. 10.3390/antiox8080272 PMC6719996 31382599

[CIT0034] Siegel RL, Miller KD, Fedewa SA, et al. (2017). Colorectal cancer statistics, 2017. CA Cancer J Clin 67:177–93. 28248415 10.3322/caac.21395

[CIT0035] Syam S, Abdelwahab SI, Al-Mamary MA, Mohan S. (2012). Synthesis of chalcones with anticancer activities. Molecules 17:6179–95. 22634834 10.3390/molecules17066179 PMC6268294

[CIT0036] Taratula O, Kuzmov A, Shah M, et al. (2013). Nanostructured lipid carriers as multifunctional nanomedicine platform for pulmonary co-delivery of anticancer drugs and siRNA. J Control Release 171:349–57. 23648833 10.1016/j.jconrel.2013.04.018 PMC3766401

[CIT0037] Thang L, Hanh N, Duong D. (2017). Study on cause-effect relations and optimization of exemestane-loaded nanostructured lipid carriers. Int J Pharm Pharm Sci 9:68–74.

[CIT0038] Yang XY, Li YX, Li M, et al. (2013). Hyaluronic acid-coated nanostructured lipid carriers for targeting paclitaxel to cancer. Cancer Letters 334:338–45. 22776563 10.1016/j.canlet.2012.07.002

[CIT0039] Yingchoncharoen P, Kalinowski DS, Richardson DR. (2016). Lipid-based drug delivery systems in cancer therapy: what is available and what is yet to come. Pharmacol Rev 68:701–87. 27363439 10.1124/pr.115.012070 PMC4931871

[CIT0040] Zhang W, Li X, Ye T, et al. (2013). Design, characterization, and in vitro cellular inhibition and uptake of optimized genistein-loaded NLC for the prevention of posterior capsular opacification using response surface methodology. Int J Pharm 454:354–66. 23876384 10.1016/j.ijpharm.2013.07.032

